# The Consequences of Preterm Birth and Chorioamnionitis on Brainstem Respiratory Centers: Implications for Neurochemical Development and Altered Functions by Inflammation and Prostaglandins

**DOI:** 10.3389/fncel.2018.00026

**Published:** 2018-02-01

**Authors:** Vanesa Stojanovska, Suzanne L. Miller, Stuart B. Hooper, Graeme R. Polglase

**Affiliations:** ^1^The Ritchie Centre, Hudson Institute of Medical Research, Melbourne, VIC, Australia; ^2^Department of Obstetrics and Gynaecology, Monash University and Hudson Institute of Medical Research, Melbourne, VIC, Australia

**Keywords:** chorioamnionitis, brainstem respiratory centers, preBötzinger complex, prostaglandins, preterm birth, apnea

## Abstract

Preterm birth is a major cause for neonatal morbidity and mortality, and is frequently associated with adverse neurological outcomes. The transition from intrauterine to extrauterine life at birth is particularly challenging for preterm infants. The main physiological driver for extrauterine transition is the establishment of spontaneous breathing. However, preterm infants have difficulty clearing lung liquid, have insufficient surfactant levels, and underdeveloped lungs. Further, preterm infants have an underdeveloped brainstem, resulting in reduced respiratory drive. These factors facilitate the increased requirement for respiratory support. A principal cause of preterm birth is intrauterine infection/inflammation (chorioamnionitis), and infants with chorioamnionitis have an increased risk and severity of neurological damage, but also demonstrate impaired autoresuscitation capacity and prevalent apnoeic episodes. The brainstem contains vital respiratory centers which provide the neural drive for breathing, but the impact of preterm birth and/or chorioamnionitis on this brain region is not well understood. The aim of this review is to provide an overview of the role and function of the brainstem respiratory centers, and to highlight the proposed mechanisms of how preterm birth and chorioamnionitis may affect central respiratory functions.

## Introduction

Preterm birth, defined as childbirth <37 weeks gestation, is a leading cause of neonatal morbidity and mortality worldwide (Lawn et al., 2005; Beck et al., 2010; Liu et al., 2012). There are ~15 million preterm births annually, of which ~1.1 million neonates die from various complications; the highest cause of neonatal mortality worldwide (WHO, 2012). The causes of preterm birth are multifaceted, ranging from environmental, fetal or maternal abnormalities/compromise (Beck et al., 2010; Haas, 2011). Importantly, more than 60% of preterm infants <28 weeks gestation are exposed to chorioamnionitis (Lahra et al., 2007), making it the most prevalent antecedent of preterm delivery.

The transition from intrauterine to extrauterine life relies on immediate and complex adaptations to effectively shift from maternal dependence to newborn physiological autonomy. In the first instance, a primary physiological driver for successful extrauterine transition is lung aeration (Hooper et al., 2015). Extensive adaptations such as the clearance of lung liquid, sufficient surfactant production, as well as changes in cardiovascular resistance and flow occur upon birth (Hillman et al., 2012). At this time, the brainstem must be fully functional as it contains vital respiratory centers that generate rhythm and coordinate breathing biomechanics (Garcia et al., 2011; Smith et al., 2013). Thus, the intrauterine to extrauterine transition is critical for neonates, and can have significant health consequences if this process is suboptimal.

Preterm infants frequently present with respiratory distress syndrome upon delivery (Fraser et al., 2004; Gallacher et al., 2016). Respiratory distress syndrome has been attributed to immature lung development, insufficient lung liquid clearance, and surfactant deficiency, which leads to poor respiration, apnoeic episodes and inadequate gas exchange (Fraser et al., 2004; Moss, 2006; Miall and Wallis, 2011; Polglase et al., 2014). As a result, preterm infants often require respiratory support following delivery, and subsequent ventilation upon transfer to the intensive care unit. Further, preterm infants exposed to infection/inflammation (chorioamnionitis) have a greater requirement for respiratory support and increased risk and severity of neurological damage than those not exposed to chorioamnionitis (Grether and Nelson, 1997; Bell and Hallenbeck, 2002; Duncan et al., 2002; Yanowitz et al., 2002; Mallard et al., 2003; Nitsos et al., 2006; Speer, 2006; Shatrov et al., 2010; Polglase et al., 2012; Galinsky et al., 2013). Despite extensive research demonstrating the link between systemic inflammation with intraventricular hemeorrhage, post-hemeorrhagic hydrocephalus and periventricular leukomalacia in the preterm brain (Heep et al., 2003; Hansen-Pupp et al., 2005; Kaukola et al., 2006; Moscuzza et al., 2011; Barton et al., 2014), the effects on the brainstem respiratory centers which regulate breathing remain largely unknown.

## Neural Control of Respiration

**Figure 1:**
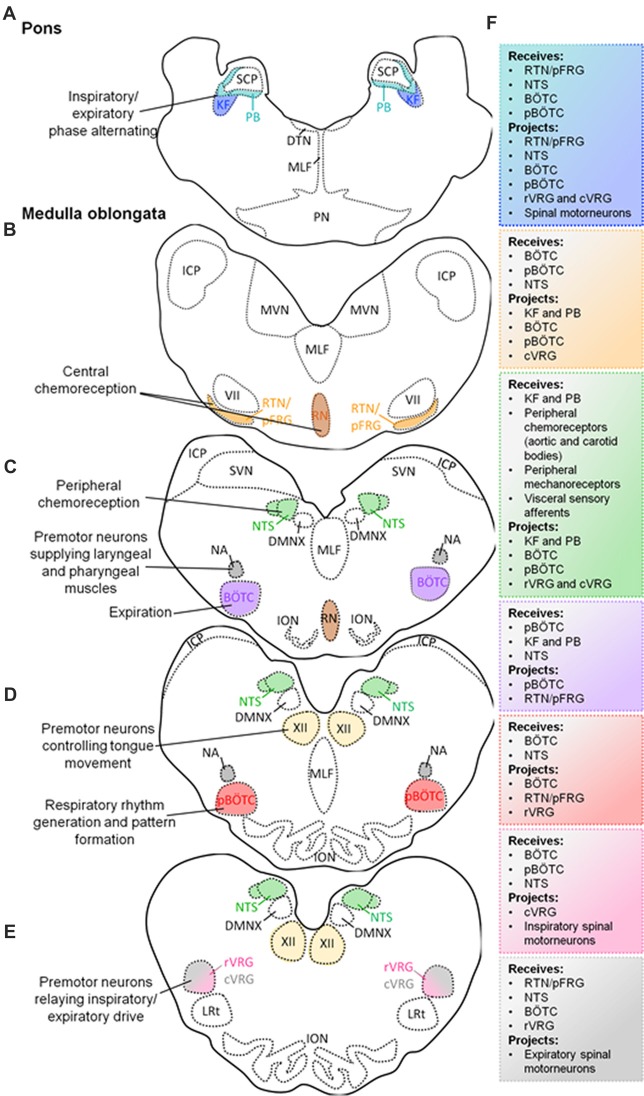
Schematic diagram of transverse sections through the brainstem exposing the main respiratory centers. Localization of the Kölliker-Fuse (KF) and parabrachial (PB) nuclei in the pons, and their simplified functions **(A)**. Localization of medullary respiratory centers: retrotrapezoid nucleus (RTN)/parafacial respiratory group (pFRG) nucleus and raphè nucleus (RN) **(B)**; nucleus tractus solitarius (NTS), Bötzinger complex (BÖTC), nucleus ambiguus (NA; **C**); pre-BÖTC (pBÖTC), hypoglossal nucleus (XII; **D**); rostral ventral respiratory group (rVRG), caudal ventral respiratory group (cVRG; **E**), and their simplified functions. Proposed neural circuitry of the brainstem respiratory centers outlining potential interactions (receiving information from, and projecting to) within, and from the brainstem **(F)**.

The neural circuitry responsible for generating and regulating eupneic respiratory rhythm are located within the brainstem (Rybak et al., 2007; Smith et al., 2009; Garcia et al., 2011). Eupneic breathing occurs in a three-phase rhythm consisting of inspiration, post-inspiration and active expiration (Smith et al., 2013). These respiratory phases are highly conserved in mammals and rely on the optimal functioning of central respiratory centers within the brainstem, which generate breathing rhythm, process and adapt to central and peripheral chemosensory information, as well as receiving afferent information from pulmonary stretch receptors, and provides and coordinates efferent innervation to the motor nerves and muscles supplying the lungs. In addition, these brainstem respiratory centers provide the neural drive for upper airway muscles important for maintaining airway patency. There are several pontomedullary respiratory centers distributed throughout the brainstem, which are critical for eupneic breathing (Figure 1).

### Pontine Respiratory Centers

The dorsolateral pons contains the Kölliker-Fuse (KF) nucleus and the parabrachial (PB) complex (Alheid et al., 2004). Neurons from the KF nucleus and the PB complex can modulate respiratory phase alternation (inspiration, post-inspiration, active expiration) through synaptic inputs to medullary nuclei (Cohen and Shaw, 2004; Dutschmann and Herbert, 2006; Ezure and Tanaka, 2006; Martelli et al., 2013; Forster et al., 2014).

### Medullary Respiratory Centers

The medulla oblongata comprises a collection of nuclei that are categorized into ventral and dorsal respiratory groups. The ventral respiratory groups consist of the pre-Bötzinger complex (pBÖTC), BÖTC, retrotrapezoid nucleus (RTN)/parafacial respiratory group (pFRG), as well as the rostral and caudal ventral respiratory groups (cVRGs; Stornetta, 2008; Smith et al., 2009; Garcia et al., 2011). The dorsal respiratory group is comprised of the nucleus tractus solitarius (NTS).

Several studies have identified the pBÖTC located within the rostral medulla oblongata to be an essential site for respiratory rhythmogenesis (Smith et al., 1991; Ramirez et al., 1998; McKay et al., 2005). Neurons within the pBÖTC are capable of spontaneous, oscillating pace-maker-like activity which is imperative for respiratory rhythmogenesis and inspiratory drive (Smith et al., 1991; Butera et al., 1999; Koshiya and Smith, 1999; Morgado-Valle et al., 2010; Phillips et al., 2016).

The BÖTC is located within the rostral ventrolateral column of the medulla oblongata and predominantly contains inhibitory expiratory neurons (Fortuna et al., 2008). The inhibitory activity of Bötzinger neurons is important for proper phase-switching from inspiratory and expiratory activities (Smith et al., 2007, 2009).

The RTN/pFRG located in the rostral medulla oblongata contains central chemoreceptors that drive respiration in a CO_2_-dependant manner (Mulkey et al., 2004; Smith et al., 2006; Stornetta et al., 2006; Guyenet and Mulkey, 2010). RTN neurons respond to local tissue acidification (high extracellular CO_2_, or its proxy, hydrogen ions), paracrine influences (ATP) by pH-sensitive astrocytes, as well as inputs from peripheral chemoreceptors (carotid and aortic bodies; information propagated by the NTS; Mulkey et al., 2004; Takakura et al., 2006; Guyenet et al., 2009; Lazarenko et al., 2009; Gourine et al., 2010; Huckstepp et al., 2010). Furthermore, the caudal raphè contains serotoninergic neurons which have demonstrated chemosensory properties (Smith et al., 2009, 2013). These neurons provide input to the RTN/pFRG, as well as the ventral respiratory column.

The rostral ventral respiratory group (rVRG) is largely comprised of excitatory inspiratory premotor neurons which provide neural input to spinal phrenic and intercostal motorneurons that innervate the diaphragm (Smith et al., 2009, 2013). The cVRG also contains excitatory expiratory premotor neurons which relay information to spinal thoracic and lumbar motorneurons innervating the lungs (Stornetta, 2008; Smith et al., 2013). In addition to the rVRG and cVRG, the medulla oblongata contains other motor nuclei involved in respiratory-related functions. These motor nuclei include the hypoglossal nucleus (XII), and the nucleus ambiguus (NA). The XII nucleus is comprised of motor neurons supplying the tongue, and plays an important role in positioning this muscle (Bailey and Fregosi, 2004; Gestreau et al., 2005; Moore et al., 2014). Modulating the position of the tongue is particularly important for maintaining upper airway patency which is critical for acquiring oxygen. Moreover, the NA contains premotor laryngeal and pharyngeal motor neurons. These neurons innervate the larynx and pharynx, through which they are involved in maintaining glottal patency important for the flow of oxygen (Smith et al., 2009; Ludlow, 2015).

The dorsal respiratory group within the medulla oblongata is comprised of the NTS which contains second-order neurons that receive and process visceral sensory information conveyed by vagal afferent nerves (Smith et al., 2009; Zoccal et al., 2014). The NTS receives and integrates chemosensory information from aortic and carotid bodies, sensory information from slow-adapting and rapid-adapting pulmonary stretch receptors, and bronchopulmonary C-fibers (Machado et al., 2000; Kubin et al., 2006). Therefore, the NTS propagates peripheral chemosensory information to all major respiratory centers within the brainstem to evoke appropriate respiratory responses.

These respiratory centers develop early in gestation and continue to mature throughout pregnancy, as evidenced by the maturation of fetal breathing movements (FBMs).

## Fetal Breathing Movements

Although the placenta is the site for respiratory gas exchange *in utero*, FBMs are detectable at 10 weeks gestational age (Boddy and Mantell, 1973; LoMauro and Aliverti, 2016). Whilst FBMs do not play a role in gas exchange, they do regulate the degree of lung liquid within the developing lungs, which is critical for normal tissue development and maturation, maintenance of intraluminal pressure and lung liquid volume, as well as the priming and entrainment of the respiratory muscles and neural circuitry for effective postnatal breathing (Harding, 1997; Baguma-Nibasheka et al., 2012; Koos and Rajaee, 2014). Contractions of the diaphragm, intercostal, and laryngeal muscles carry out the physical task of FBMs, however, these muscles are under the control of the brainstem respiratory centers, which generate and coordinate breathing patterns (Dawes, 1984; Harding, 1997; Greer et al., 2006; LoMauro and Aliverti, 2016).

The incidence of FBMs increases and becomes more episodic (Dawes et al., 1970, 1972; Bowes et al., 1981; Dawes, 1984) with gestational age from about 2% of time at 10 weeks, 6% at 19 weeks, 13.7% at 24–26 weeks (Natale et al., 1988), 14.2% at 26–28 weeks (Natale et al., 1988) and to 31% of the time at 30 weeks. Between 30 weeks and 40 weeks, the mean incidence of FBMs remains at about 30%–40%, although the differences between individual fetuses can range from 17% to 65% (Patrick et al., 1980; Bocking et al., 1982; Harding, 1994). The duration of time spent in apnea also increases, sometimes lasting for up to 2 h in humans at 38–39 weeks (Patrick et al., 1980). These changes in apnoeic periods correlate with changing/maturing patterns of FBMs which also reduce in frequency in late gestation, but increase their duration (Trudinger and Knight, 1980; Clewlow et al., 1983), mimicking breathing *post-partum*. The frequency of FBMs is highly variable between individuals. At 24–28 weeks of gestation in humans, the rate of FBMs is 42–44 breaths per minute (Natale et al., 1988), increases to 55–62 breaths per minute at 30–31 weeks of gestation (Patrick et al., 1980), and reduces again to 45–48 breaths per minute at 38–39 weeks of gestation (Patrick et al., 1980).

FBMs are also regulated by a number of physiological factors. As in the adult, increases in the partial pressure of CO_2_ (P_a_CO_2_; hypercapnia) profoundly stimulate FBMs (Connors et al., 1988), while hypocapnia reduces FBMs (Connors et al., 1988, 1989; Darnall, 2010). Acute hypoxia stimulates respiratory activity in adults, whereas it abolishes FBMs (Koos et al., 1986; LoMauro and Aliverti, 2016). Other factors which can influence FBMs include neurotransmitters, glucose (Natale, 1980), as well as inflammation and prostaglandins (Dong and Feldman, 1995; Herlenius et al., 1997; Olsson et al., 2003; Siljehav et al., 2014).

The changes to the ontogeny of FBMs throughout gestation reflect the maturation of the brainstem respiratory centers. However, being born preterm, particularly extremely preterm (<28 weeks), coincides not only with an immature lung, but also an immature respiratory control center.

## Preterm Birth: Potential Effects on Neurochemical Development in the Brainstem and Respiratory-Related Activity

Whilst autonomous neonatal breathing is critical for sustaining life, the brainstem respiratory centers are not completely mature at birth, and instead, continue to develop and refine postnatally (Wong-Riley and Liu, 2008). It is essential that there is sufficient prenatal development of the brainstem respiratory centers to a point of “readiness” at birth in order for the neonate to generate stable and continuous breathing movements (Carroll and Agarwal, 2010).

Preterm infants have demonstrated altered/abnormal ventilatory responses to hypoxic and hypercapnic conditions (Zhao et al., 2011). In hypoxic circumstances, preterm infants display an initial (albeit transient) rise in respiratory rate, alongside an increase in tidal volumes. However, this initial increase in respiration is then followed by a continuous decline in spontaneous breathing (“hypoxic ventilatory depression”) which can last for several weeks, and is thought to play a role in delayed respiratory adjustments in postnatal life (Nock et al., 2004; Zhao et al., 2011). Ventilatory responses to hypercapnia by preterm infants are poor. Typical responses by preterm infants include prolonged expiration times, and a lack of increased respiratory rate, and low/insufficient tidal volumes. Consequently, this altered hypercapnic response results in low minute ventilation (Zhao et al., 2011). These insufficient hypoxic and hypercapnic responses likely reflect the immaturity of the brainstem and/or altered neuronal functions.

During the first 2 weeks of postnatal life the brainstem respiratory centers undergo dynamic developmental changes which have been associated with a large degree of neuronal maturation and neurochemical modifications (mostly studied in rodents; Wong-Riley and Liu, 2005). Preterm infants are not only forced to sustain continuous breathing movements with immature neural control (neuronal hypoplasia, poor synaptic connections and myelination), but the typical postnatal adjustments of neurotransmitters may not follow the same course as it would in full-term neonates (Stokowski, 2005). There are limited data on the neurochemical changes in the fetal brainstem as most studies have focused on differential expression in the embryonic and postnatal stages of development.

Glutamate is the principal neurotransmitter that drives inspiratory activity and the processing of sensory inputs, and its expression generally increases in rats postnatally, particularly in the pBÖTC and the NTS (Liu and Wong-Riley, 2005; Benarroch, 2007). In addition, studies in rats have shown that the expression of glutaminergic receptors (N-methyl-D-aspartate; NMDA; α-amino-3-hydroxy-5-methyl-4-isoxazoleproprionc acid (AMPA); and metabotropic glutaminergic receptors) also increase with age (Liu and Wong-Riley, 2005; Wong-Riley and Liu, 2005). It is unclear how preterm birth can impact the postnatal fluctuations in glutamate expression within the brainstem respiratory centers, and how this is associated with irregular neonatal breathing. But presumably, weak/immature glutaminergic synaptic transmission would alter chemosensory input, and breathing rhythmogenesis and pattern formation.

Gamma-aminobutyric acid (GABA) and glycine are inhibitory neurotransmitters which modulate inspiratory activity to allow for the proper transition to the expiratory phase. The developmental expression of GABA in rats has been shown to steadily decrease in the NTS, but increase in the pBÖTC (Liu and Wong-Riley, 2005), whereas glycine receptor immunoreactivity has been shown to increase with age in the pBÖTC and the NTS (Liu and Wong-Riley, 2005). In late embryonic and early postnatal life, GABAergic and glycinergic neurotransmission can be excitatory until a developmental shift from depolarizing to hyperpolarizing activity occurs. Preterm neonates display an enhanced sensitivity to GABA (amongst other neurotransmitters and neuromodulators including adenosine, serotonin, and prostaglandins) which can lead to respiratory depression (Martin et al., 2004; Zhao et al., 2011). Whether this respiratory depression is due to excessive excitatory or inhibitory activity remains to be determined. The effects of preterm birth on the proper switching of GABAergic and glycinergic neurotransmission (excitatory to inhibitory) in the brainstem respiratory neurons remains to be investigated.

Central chemosensitivity to CO_2_ within the brainstem is primarily mediated by cholinergic, serotoninergic, and glutaminergic neurotransmission. Acetylcholine is a neurotransmitter acting post-synaptically on inspiratory neurons within the pBÖTC (Burton and Kazemi, 2000; Lai et al., 2001; Shao and Feldman, 2009). It is currently unclear how preterm birth can affect the maturation of cholinergic neurons within brainstem respiratory centers, but this would presumably affect signaling of central chemosensory information. Serotoninergic neurons in the ventral surface of the medulla oblongata and the raphè nucleus (RN) also respond to elevated CO_2_ and changes in blood pH (Richerson et al., 2001; Hilaire et al., 2010). These chemosensitive neurons continue to develop postnatally (as evidenced in rats; Davis et al., 2006). Substance P is a neurotransmitter that can evoke inspiratory activity and propagate chemosensory information through its binding to neurokinin-1 receptors which are strongly expressed on rhythmogenic pre-Bötzinger neurons and central chemoreceptors in the RTN/pFRG (Gray et al., 2001; Nattie and Li, 2002; Shvarev et al., 2002). Research investigating the effects of preterm birth on serotoninergic and substance P-expressing neurons in the brainstem respiratory nuclei are lacking, but impaired development and/or maturation would presumably lead to inadequate breathing adaptations due to poor signaling to the pBÖTC, and other respiratory-related nuclei.

Adenosine is a neurotransmitter which exerts strong inhibitory activity of inspiratory neurons, and ultimately decreases inspiratory drive to phrenic motorneurons innervating the diaphragm (Dong and Feldman, 1995; Herlenius et al., 1997). Postnatal changes of adenosine expression in the brainstem respiratory centers remain unclear, and it is unknown how exactly preterm birth may affect the expression of this neurotransmitter or its respective receptors. However, it is well-established that preterm infants are particularly sensitive to adenosine, and this suggests that adenosine receptors have developed and matured to some degree.

Whilst there are some limited data describing the developmental changes in the brainstem respiratory centers, the effects of preterm birth remain incompletely understood. Additionally, it is well established that inflammation can alter neuronal function, and thus, delineating the effects of preterm birth and chorioamnionitis on the brainstem respiratory centers is challenging.

## Chorioamnionitis

Infection and inflammation of the chorionic membrane and/or the amnion/amniotic fluid collectively refers to chorioamnionitis. This inflammatory condition is most commonly caused by a maternal ascending polymicrobial infection, and can be contracted by the fetus following exposure to amniotic fluid, or via placental-fetal circulation (Galinsky et al., 2013). A myriad of bacterial, viral and fungal species have been implicated in the pathogenesis of chorioamnionitis, with ureaplasmas being the most common organism isolated (Viscardi, 2010; Sweeney et al., 2016). Given the delayed diagnosis of chorioamnionitis (usually placental pathology days after birth), and insufficient therapeutic intervention to reduce inflammatory insult during gestation, the vital organs of the fetus can be significantly damaged.

Chorioamnionitis is known to induce “fetal inflammatory response syndrome”, which is characterized by systemic inflammation, notably injuring the lungs and the brain (Polglase et al., 2012; Galinsky et al., 2013; Kallapur et al., 2014). Research has demonstrated that chorioamnionitis/lipopolysaccharide (LPS)-induced infection is strongly associated with inflammatory and hypoxia-mediated brain injury, observed in both infants and rodents (Yoon et al., 2000; Shalak et al., 2002; Grether et al., 2003; Yang et al., 2005; Dessardo et al., 2012; Ecevit et al., 2014). Chorioamnionitis can lead to neurodevelopmental abnormalities, and poor cognitive, behavioral and neuromotor outcomes in infants, and is implicated in 11%–22% of cerebral palsy (Murphy et al., 1995; Wu, 2002; Inder et al., 2003; Wu et al., 2003; Shatrov et al., 2010).

Furthermore, it is well known that infection and inflammation have the capacity to alter autoresuscitation ability and induce apnoeic episodes in neonates, as well as in rodents and piglets (Frøen et al., 2000; Kamaluddeen et al., 2009; Stock et al., 2010; Herlenius, 2011; Lorea-Hernández et al., 2016). Thus, it is unsurprising that chorioamnionitis is linked to sudden infant death syndrome, recurrent apnea in preterm infants, and obstructive sleep apnea syndrome in ex-preterm children (Kaufman and Fairchild, 2004; Weber et al., 2008; Zhao et al., 2011; Simonsen et al., 2014; Tapia et al., 2016).

Antenatal corticosteroids are currently used to accelerate lung maturation in preterm infants (Surbek et al., 2012; Freeman et al., 2015). Corticosteroids have also proven effective in reducing the severity of histological chorioamnionitis and minimizes respiratory distress syndrome (Surbek et al., 2012; Freeman et al., 2015). In addition, antenatal corticosteroids have also shown protective effects against necrotizing enterocolitis, intraventricular hemorrhage, major brain lesions, and periventricular leukomalacia (Surbek et al., 2012; Freeman et al., 2015). Studies investigating the effects of single or repeated corticosteroid use on auditory brainstem responses in preterm infants and animal models with and without chorioamnionitis have revealed conflicting results (Amin et al., 2003; Amin and Guillet, 2007; Church et al., 2012). Few human studies have demonstrated that corticosteroids have no effect on auditory brainstem responses (Amin et al., 2003; Amin and Guillet, 2007). Whereas in rats, repeated courses of corticosteroids negatively affected neural transmission time and auditory brainstem pathways (Church et al., 2012). Furthermore, repeated corticosteroid administration in fetal sheep leads to a reduction in cerebral weight without affecting the cerebellum and brainstem (Huang et al., 1999). Moreover, the effects of corticosteroids on the brainstem respiratory centers remain unknown. The exact mechanisms underlying the changes in respiratory function are not entirely understood, but emerging work highlights key roles for pro-inflammatory cytokines and prostaglandins in depressing respiratory function.

### Mechanisms of Chorioamnionitis and Brainstem Function

Chorioamnionitis can be experimentally induced in animal models through intra-amniotic injections of LPS (cell wall constituent of gram-negative bacteria; Polglase et al., 2012; Barton et al., 2014; Ireland et al., 2015). This bacterial endotoxin gives robust and reproducible inflammatory responses, and as such, is widely used to experimentally induce chorioamnionitis.

**Figure 2:**
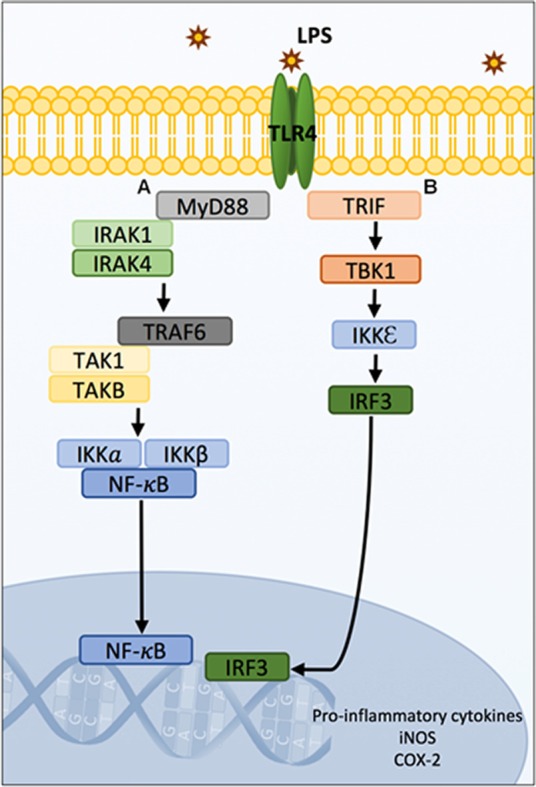
Schematic diagram of TLR4 signaling pathways. MyD88 and TRIF-mediated LPS/TLR4 downstream signaling pathways leading to gene transcription of pro-inflammatory cytokines, iNOS and COX-2. The LPS/TLR4 signal transduction pathways are typically divided into MyD88-dependent and independent cascades. Following LPS stimulation, IRAK1 and IRAK4 are recruited to the MyD88-dependant pathway **(A)** and interact with TRAF6 proteins. TRAF6 recruits TAK1 and TABs which activate the NF-κB and/or MAPK. In resting states, NF-κB is sequestered in the cytosol by IKKα and IKKβ. Phosphorylation of the IKK complexes by TAK1 results in their proteasomal degradation and liberation of NF-κB which subsequently translocates from the cytosol to the nucleus where it can induce gene expression. Concurrently, TAK1 activates the MAPK pathway resulting in the phosphorylation and AP-1 which translocates to the nucleus and binds to DNA. Additionally, the LPS/TLR4 MyD88-independent signaling pathway involves the activation of TRIF **(B)** and signaling to TBK1, IKK and IRF3. This pathway results in interferon-related cytokines, and can potentiate NF-κB gene transcription. Ultimately, gene transcription leads to the production of IL-1β, IL-6, IL-8, TNF-α, TNF-β, iNOS and COX-2. Abbreviations: LPS, Lipopolysaccharide; TLR4, Toll-like receptor 4; MyD88, myeloid differentiation primary response gene 88; IRAK1 and IRAK4, interleukin 1-associated kinases-1 and 4; TRAF6, tumor necrosis factor associated factor 6; TAK1, transforming growth factor-β-activated kinase-1; TABs, TAK1-binding proteins; MAPK, mitogen-activated protein kinases; IKKα and IKKβ, inhibitory IkB kinases; AP-1, activation of the transcription factor activator protein 1; TRIF, TIR-domain-containing adapter-inducing interferon-β; TBK1, TANK-binding kinase; IRF3, IKK, and interferon regulatory factor 3; iNOS, inducible nitric oxide synthase; COX-2, cyclooxygenase 2.

LPS is a ligand for toll-like receptors (TLRs, namely TLR4), which stimulate downstream signaling pathways leading to pro-inflammatory cytokine production (Beutler, 2003; Lu et al., 2008). The activated TLR4 pathway results in interferon-related cytokines, and can potentiate NF-κB gene transcription (Pålsson-McDermott and O’Neill, 2004). Ultimately, gene transcription leads to the production of IL-1β, IL-6, IL-8, TNF-α, TNF-β, inducible nitric oxide synthase (iNOS), and inducible cyclooxygenase2 (COX-2; mediates prostaglandin synthesis; Figure 2; Blackwell and Christman, 1997; Poligone and Baldwin, 2001; Tak and Firestein, 2001; Aktan, 2004). In the brain, microglia and astrocytes express TLRs and can also play a major role in cytokine production (Kielian, 2006).

#### Inflammatory Cytokines and Effects on the Brainstem

LPS exposure upregulates IL-1β and IL-6 mRNA expression within the brainstem of rat pups (Balan et al., 2012), which may in turn alter neuronal function within the pBÖTC. Electrophysiological recordings of pre-Bötzinger neurons from neonatal mice following intrauterine LPS administration has revealed functional changes of pacemaker neurons that are characterized as large amplitude bursts, at slow and irregular firing frequency (Ramirez et al., 2016). If IL-1β and IL-6 depress inhibitory synaptic transmission, and simultaneously elevate excitatory signaling in the pBÖTC, then this could explain the prolonged inspiratory drive and the absent expiratory activity that leads to apnea. Neuronal activity can be rapidly and differentially modulated by cytokines, and these functional changes may persist long-term (Vezzani and Viviani, 2015). The pro-inflammatory cytokines IL-1β, IL-6 and TNF-α can modulate neuronal function within the central nervous system, particularly by potentiating excitatory signaling, and depressing inhibitory transmission (Galic et al., 2012). These cytokines alter neuronal excitability through post-translational modifications of glutaminergic, GABAergic, and glycinergic receptors, ultimately affecting neurotransmission and synaptic plasticity (Galic et al., 2012; Vezzani and Viviani, 2015). Studies in rodents and in *in vitro* hippocampal neurons demonstrate that IL-1β alters neuronal function in a concentration-dependent manner; at low concentrations IL-1β inhibits voltage-gated calcium currents, and lowers intracellular calcium concentrations (thereby decreasing neurotransmitter release), whereas high concentrations of IL-1β increase ionotropic glutamate receptor expression (NMDA), and decreases transmission at GABAergic and glycinergic receptors in rat hippocampal and cerebellar cultures (Campbell and Lynch, 1998; Wang et al., 2000; Viviani et al., 2003; Galic et al., 2012). TNF-α alters neuronal excitability through the upregulation of NMDA and AMPA receptor expression, and induces GABA receptor endocytosis, as observed in the rat hippocampus and cerebellum (Beattie et al., 2002; Fourgeaud and Boulanger, 2010; Galic et al., 2012). This leads to an increase in excitatory output (and possibly excitotoxicity), as well as a decrease in inhibitory signaling. IL-6 has been shown to play both protective and destructive roles within the central nervous system. Research has shown that IL-6 decreases metabotropic glutamate receptor expression, but can also excessively activate NMDA receptors and induce excitotoxicity (D’Arcangelo et al., 2000; Conroy et al., 2004; Vereyken et al., 2007; Wang et al., 2007). Additionally, IL-6 has also demonstrated the capacity to decrease GABAergic and glycinergic neurotransmission in dorsal horn neurons of rat spinal cord (Kawasaki et al., 2008).

**Figure 3:**
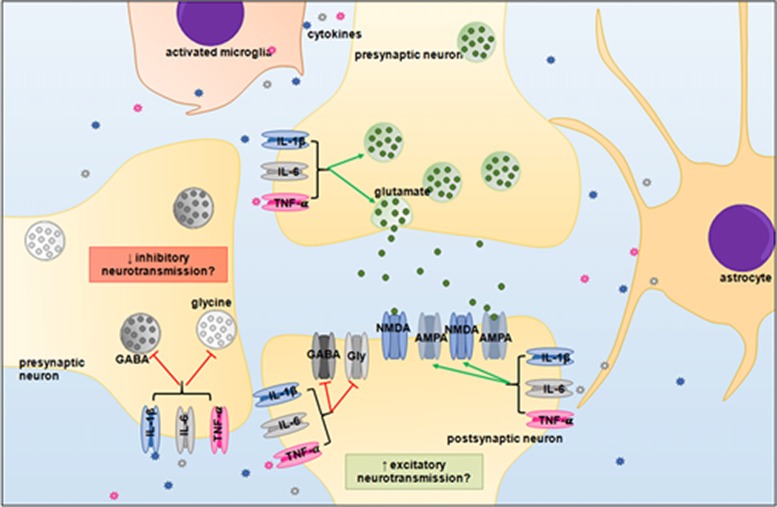
Proposed neuromodulatory effects of pro-inflammatory cytokines in the brainstem. IL-1β, IL-6 and TNF-α may upregulate glutaminergic receptor expression and potentiate excitatory signaling, whilst simultaneously depressing inhibitory GABAergic and glycinergic neurotransmission. An imbalance between excitatory and inhibitory signaling could desynchronize the neural circuitry of the brainstem respiratory centers. Abbreviations: GABA, gamma-Aminobutyric acid; Gly, glycine; NMDA, N-methyl-D-aspartate; AMPA, α-amino-3-hydroxy-5-methyl-4-isoxazoleproprionc acid.

It is now well established that LPS-induced systemic infection/inflammation can alter breathing frequency and chemosensory responses, however the mechanisms underlying the changes in respiratory functions remain unclear (Huxtable et al., 2011). An imbalance between excitatory and inhibitory neurotransmission has been demonstrated previously in the brainstem of rats and piglets during hypoxic conditions (Kazemi and Hoop, 1991; Huang et al., 1994; McCormick et al., 1998; Hoop et al., 1999), but less is known about the effects of inflammation on neurotransmitters within the brainstem. As neurons within the brainstem respiratory centers utilize glutamate, GABA and glycine, then pro-inflammatory cytokines could affect the balance of excitatory and inhibitory neurotransmitters, and thus, may alter neuronal function and respiratory responses (Figure 3).

Systemic administration of the pro-inflammatory cytokines IL-1β, IL-6 and TNF-α induces their own mRNA expression within the rat NTS (Churchill et al., 2006). IL-1β injection directly into the rat NTS increases inspiratory time as early as 20 min-post application, which is associated with delayed expiratory activity (observed after 80 min; Gresham et al., 2011). Furthermore, the systemic administration of IL-1β leads to decreased respiratory frequency and induces apneustic episodes (Gresham et al., 2011). It is currently unclear whether IL-1β induces changes in excitatory neurotransmission of the NTS to modulate respiratory rhythm, or whether it does so by other downstream effects. Strong IL-1β-immunoreactivity is observed in the rat NTS and area postrema following LPS exposure (Balan et al., 2011, 2012). Previous work has shown that the increase in IL-1β mRNA expression within the NTS can be abrogated by vagotomy (Balan et al., 2012).

Intraperitoneal and intrapulmonary injection of IL-1β to mouse and rat pups attenuates hypercapnic and hypoxic responses (Balan et al., 2012; Siljehav et al., 2014), indicative that chemosensory reflexes are impaired in response to inflammation. It is unclear whether altered chemosensory responses are due to compromised central chemoreceptor functions exclusively (RTN/pFRG neurons), or whether there are changes to vagal afferent signaling to the NTS, and/or impaired propagation of peripheral chemosensory information to brainstem respiratory centers by second-order NTS neurons. It is likely that infection/inflammation would induce considerable changes to both central and peripheral chemoreflexes.

In addition to IL-1β expression in the NTS, strong immunoreactivity is also observed in the area postrema following LPS exposure (Balan et al., 2011, 2012). Most research to date has attributed the loss of blood brain barrier integrity as the main pathway for brain inflammation and injury following systemic endotoxin or cytokine exposure. However, the NTS forms connections with the area postrema which is a circumventricular region of the brainstem that could be another entrance for peripheral inflammation.

LPS administration induces c-Fos immunoreactivity in neurons from the rostral ventrolateral medulla, NTS, and the KF/PB nuclei in the rat (Zhang et al., 2000). This suggests that neurons from these respiratory nuclei are responsive to infection/inflammation, but the exact mechanisms for how (and which) inflammatory mediators affect the functions of respiratory-related neurons remain unclear.

#### Prostaglandins and Effects on the Brainstem

In addition to gene transcription of pro-inflammatory cytokines, it is well-established that NF-κB and mitogen-activated protein kinases (MAPK) signaling can enhance COX isozyme expression leading to elevated prostaglandin synthesis (Ricciotti and FitzGerald, 2011). The two main isoforms of COX are COX-1 and COX-2. COX-1 is constitutively expressed, whilst COX-2 is induced upon tissue injury and inflammation. COX isozymes convert arachidonic acid to the precursor substrate prostaglandin H2 (PGH_2_; Poligone and Baldwin, 2001; Simmons et al., 2004). PGH_2_ is utilized for the synthesis of prostaglandin E2 (PGE_2_), prostaglandin D2, prostaglandin F2α, and prostacyclin (Ricciotti and FitzGerald, 2011). Specifically, microsomal prostaglandin E2 synthase-1 (mPEGS-1) catalyzes the synthesis of PGE_2_ from PGH_2_ (Murakami et al., 2000; Bahia et al., 2014). There is increasing evidence that COX-2-mediated PGE_2_ production plays a role in inflammation-induced preterm and neonatal brain injury, and altered function of respiratory-related neurons (Malaeb and Dammann, 2009; Fathali et al., 2010; Strunk et al., 2014; Jin et al., 2015). Similar to pro-inflammatory cytokines, PGE_2_ can alter neuronal excitability and neurotransmission. PGE_2_ has been shown to both enhance and inhibit glutaminergic transmission, depress glycingeric signaling, and modulate GABAergic receptor expression, and although the exact mechanisms remain unclear, neuromodulation is thought to be dependent on the type of eicosanoid prostaglandin PGE2 receptors (EPRs) stimulated (Ahmadi et al., 2002; Chen and Bazan, 2005; Laaris and Weinreich, 2007; Marty et al., 2008; Lin et al., 2014; Yang et al., 2015).

PGE_2_ has been associated with irregular breathing movements *in vivo*, as well as inhibitory effects on brainstem centers generating respiratory rhythm and chemosensory responses *in vitro* in slices generated from rat and mouse pups (Hofstetter et al., 2007; Siljehav et al., 2014; Forsberg et al., 2016). Moreover, PGE_2_ has been shown to depress FBMs, induce hypoventilation, reduce respiratory frequency and cause apnea in fetal sheep (Kitterman et al., 1983; Guerra et al., 1988). When directly injected into the mouse pBÖTC at a low concentration (<200 nM), PGE_2_ increases sigh frequency with no apparent effects on eupneic breathing (Koch et al., 2015). High concentrations of PGE_2_, however, are shown to promote eupneic breathing (Koch et al., 2015). Conflicting results suggest multiple roles for PGE_2_ in the brainstem respiratory centers, and that there may be differential effects on breathing which may be context dependent. COX inhibition by indomethacin has been shown to stimulate breathing movements in fetal sheep which strengthens the notion that prostaglandins modulate respiratory activity (Jansen et al., 1984).

**Figure 4:**
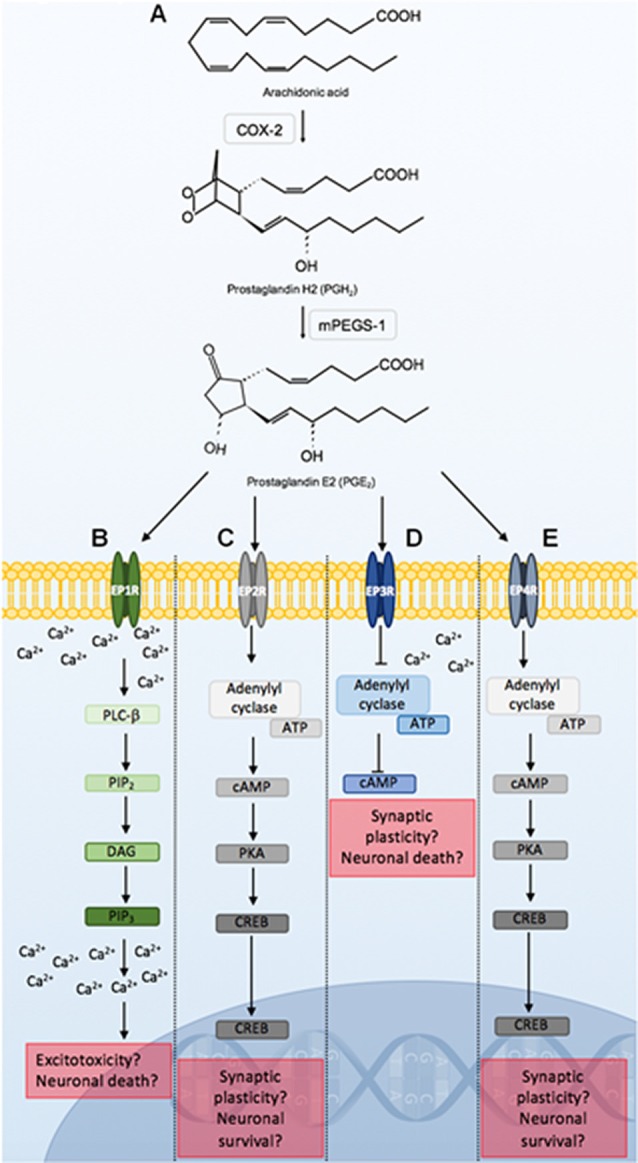
Prostaglandin E2 (PGE_2_) signaling through EPRs 1–4. COX-2 conversion of arachidonic acid to PGH_2_, is utilized by mPEGS-1 to synthesize PGE_2_ **(A)**. PGE_2_ binding to EPRs initiates distinct signaling pathways that may lead to alterations in neuronal function, or result in neuroprotection or death. PGE_2_ ligation to EP1R leads to PLC-β activation, which hydrolyzes PIP_2_, DAG and PIP_3_. PIP_3_ binds to respective receptors on the endoplasmic reticulum leading to further increases in intracellular calcium. Alterations in calcium homeostasis as a result of EP1R signaling can lead to excitotoxicity and neuronal death **(B)**. PGE_2_ binding to the EP2R initiates adenylyl cyclase activation of cAMP and PKA. PKA activates CREB which is a major transcription factor that can lead to synaptic plasticity, and neuroprotection **(C)**. PGE_2_ binding to EP3R inhibits ATP catalyzation by adenylyl cyclase, causing a reduction in cAMP, as well as an increase in intracellular calcium. This can modulate neuronal excitability and firing rate, and lead to cell death. PGE_2_ signaling through the EP4R is similar to the EP2R pathway **(D)**. PGE2 binding to EP4R results in similar signaling cascades observed following EP2R stimulation **(E)**. Abbreviations: EP1–4, eicosanoid prostanoid receptors 1–4; PLC-β, phospholipase C-β; PIP_2_, phosphatidylinositol-4,5-biphosphate; DAG, Diacylglycerol; PIP_3_, inositol-1,4,5-triphosphate; ATP, adenosine triphosphate; cAMP, cyclic adenosine monophosphate; PKA, protein kinase A; CREB, cAMP-response element binding; mPEGS-1, microsomal prostaglandin E2 synthase-1.

PGE_2_ is implicated in a number of neuropathological conditions which may be due to its capacity to bind to several G-protein coupled receptors. These include the EPRs 1–4 (EP1R, EP2R, EP3R, EP4R). Binding to these receptors can result in distinct signaling pathways (Figure 4). These signaling pathways may induce neuronal damage, dysfunction, or protection and may also potentiate or ameliorate inflammation, and alter cerebral blood flow.

PGE_2_ binding to EP1R leads to increases in intracellular calcium. Alterations in calcium homeostasis as a result of EP1R signaling has been associated with excitotoxicity and neuronal death in mice (Kawano et al., 2006; Shimamura et al., 2013). Furthermore, EP1R activation stimulates vasoconstriction which can limit cerebral blood flow and potentiate hypoxic-ischemic events. In a neonatal rat model of hypoxic-ischemic encephalopathy, a selective EP1R antagonist significantly reduces cerebral injury (Taniguchi et al., 2011). The role of EP1R in chorioamnionitis-induced brainstem injury remains unknown.

PGE_2_ binding to EP2R leads to cAMP-response element binding (CREB) activation which is a major transcription factor that can induce synaptic plasticity, and neuroprotection (Carlezon et al., 2005; Kalinski, 2011; Liang et al., 2011; Sakamoto et al., 2011). PGE_2_ binding to the EP2R has demonstrated neuroprotective effects in states of cerebral ischemia and excitotoxicity in the rat, and in mouse models (EP2R knockout, and middle cerebral artery occlusion; McCullough et al., 2004; Li et al., 2008). However, upon LPS exposure, COX-2 and iNOS-mediated neurotoxicity is abrogated by microglial EP2R deletion (Shie et al., 2005). It appears that the effects of PGE_2_ ligation to EP2R may be context dependent, but in circumstances of systemic inflammation, it is presumed that activation of the EP2R may play a deleterious role in the brainstem.

EP3R has the greatest affinity for PGE_2_ and has previously been implicated in neuroinflammation and neuronal dysfunction (Nakamura et al., 2000; Hofstetter et al., 2007; Hein and O’Banion, 2009; Leclerc et al., 2015). PGE_2_ binding to EP3R leads to a reduction in cyclic adenosine monophosphate (cAMP), and an increase in intracellular calcium which can affect neuronal excitability and firing rate (Bos et al., 2004; Mohan et al., 2012). Downstream IL-1β-mediated PGE_2_ production and subsequent binding to EP3R has been shown to reduce excitatory vagal neurotransmission to the NTS in rats (Marty et al., 2008). This would likely impact peripheral chemosensory input to the brainstem.

EP4R is highly expressed within the hypothalamus and brainstem, and functions similarly to EP2R (Andreasson, 2010; Taniguchi et al., 2014). PGE_2_ signaling through the EP4R is similar to the EP2R pathway as it offers neuroprotection in models of excitotoxic or hypoxic-ischemic injury, however, it may can also modulate pro-inflammatory and anti-inflammatory responses, as observed in the rat, and in mouse EP4R knockout studies (Zhang and Rivest, 1999; Andreasson, 2010; Shi et al., 2010). EP4R has been localized to the NTS and ventrolateral medulla of the brainstem, and its expression appears to increase following systemic IL-1β administration (Zhang and Rivest, 2000). However, further work is required to determine its role in chorioamnionitis-associated brainstem injury.

## Conclusion

Preterm birth is associated with suboptimal development of the brainstem, and subsequently reduced respiratory control. Further, chorioamnionitis is strongly associated with preterm birth, and leads to an increased risk and severity of respiratory complications. There is extensive research on chorioamnionitis-induced white matter brain injury, however, the effects of inflammation on the brainstem, which contains central respiratory centers, remains unclear. Inflammation is associated with elevated prostaglandin synthesis, and PGE_2_ specifically has been shown to cause functional changes of respiratory-related neurons within the brainstem. Dysregulation and damage to these brainstem centers may be implicated in the multifaceted pathophysiology of respiratory depression in preterm neonates. Understanding how chorioamnionitis may affect these central respiratory centers could lead to effective therapeutic interventions within the delivery room, with the goal of reducing brain injury and preserving the neural circuitry controlling rhythmic and coordinated respiratory functions.

## Author Contributions

VS: wrote the manuscript and made figures. SLM, SBH and GRP: revised the manuscript.

## Conflict of Interest Statement

The authors declare that the research was conducted in the absence of any commercial or financial relationships that could be construed as a potential conflict of interest.
